# Suberoylanilide hydroxamic acid increases anti-cancer effect of tumor necrosis factor-α through up-regulation of TNF receptor 1 in lung cancer cells

**DOI:** 10.18632/oncotarget.14628

**Published:** 2017-01-13

**Authors:** Bo Ra You, Bo Ram Han, Woo Hyun Park

**Affiliations:** ^1^ Department of Physiology, Medical School, Institute for Medical Sciences, Chonbuk National University, Jeonju, 561-180, Republic of Korea

**Keywords:** lung cancer, histone deacetylase, suberoylanilide hydroxamic acid, tumor necrosis factor-α, apoptosis

## Abstract

Suberoylanilide hydroxamic acid (SAHA) as a histone deacetylase (HDAC) inhibitor has anti-cancer effect. Here, we evaluated the effect of SAHA on HDAC activity and cell growth in many normal lung and cancer cells. We observed that the HDAC activities of lung cancer cells were higher than that of normal lung cells. SAHA inhibited the growth of lung cancer cells regardless of the inhibitory effect on HDAC. This agent induced a G2/M phase arrest and apoptosis, which was accompanied by mitochondrial membrane potential (MMP: ΔΨm) loss in lung cancer cells. However, SAHA did not induce cell death in normal lung cells. All tested caspase inhibitors prevented apoptotic cell death in SAHA-treated A549 and Calu-6 lung cancer cells. Treatment with tumor necrosis factor-alpha (TNF-α) enhanced apoptosis in SAHA-treated lung cancer cells through caspase-8 and caspase-9 activations. Especially, SAHA increased the expression level of TNF-α receptor 1 (TNFR1), especially acetylation of the region of TNFR1 promoter −223/-29 in lung cancer cells. The down-regulation of TNFR1 suppressed apoptosis in TNF-α and SAHA-treated lung cancer cells. In conclusion, SAHA inhibited the growth of lung cancer cells via a G2/M phase arrest and caspase-dependent apoptosis. SAHA also enhanced apoptotic effect of TNF-α in human lung cancer cells through up-regulation of TNFR1. TNF-α may be a key to improve anti-cancer effect of HDAC inhibitors.

## INTRODUCTION

Histone deacetylase (HDAC) triggers the suppression of transcription by removal of acetyl groups from lysine amino acid on histones [1]. Many studies have been demonstrated that the expression of HDAC is upregulated in many human cancers [2–4]. Therefore, diverse HDAC inhibitors are being studied in various cancers [5–7]. Suberoylanilide hydroxamic acid (SAHA) is the first HDAC inhibitor approved by U.S. Food and Drug Administration for the treatment of cutaneous T-cell lymphoma. It also has been clinically tested in lung and gastrointestinal cancers [8–10]. Numerous evidences indicate that SAHA induces apoptosis and inhibits metastasis in various cancer cells [11, 12].

The tumor necrosis factor (TNF) family is composed of 19 ligands and 29 receptors. It can modulate inflammation, proliferation and apoptosis in the cells [13–16]. There are three major cytokines in TNF family, TNF-α, TNF-related apoptosis-inducing ligand (TRAIL) and Fas ligand (FasL). TNF-α can bind two receptors, TNF receptor 1 (TNFR1) and TNF receptor 2 (TNFR2). When TNF-α binds to TNFR1, it induces caspase-8 activation and then finally causes apoptosis [17]. TNF-α also activates NF-κB pathway and AP-1 signaling via binding to TNFR2 [18]. TRAIL and FasL also lead to apoptosis mediated by their receptors [19]. TNF family cytokines can be a novel molecule because they sensitize cancer cells to the anti-cancer effect of HDAC inhibitor via induction of death receptor [20].

Lung cancer is the most common cancer and major cause of cancer death in the worldwide. The main types are small cell lung cancer (SCLC) and non-small cell lung cancer (NSLC). There are three subtypes of NSCLC, adenocarcinoma, squamous cell carcinoma and large cell carcinoma. The major cause of lung cancer are tobacco smoke and this environmental factor could increase the possibility of histone modification in lung cells [21]. Although SAHA has been tested clinically in lung cancer [8, 22], little is known about whether SAHA is a proper anti-cancer drug in lung cancer. Therefore, in this study, we investigated the effect of SAHA on HDAC activity and cell death, and suggested a promising combination strategy using TNF-α to improve anti-cancer effect of SAHA in lung cancer cells.

## RESULTS

### Effects of SAHA on HDAC activities in normal lung and cancer cells

**Figure 1:**
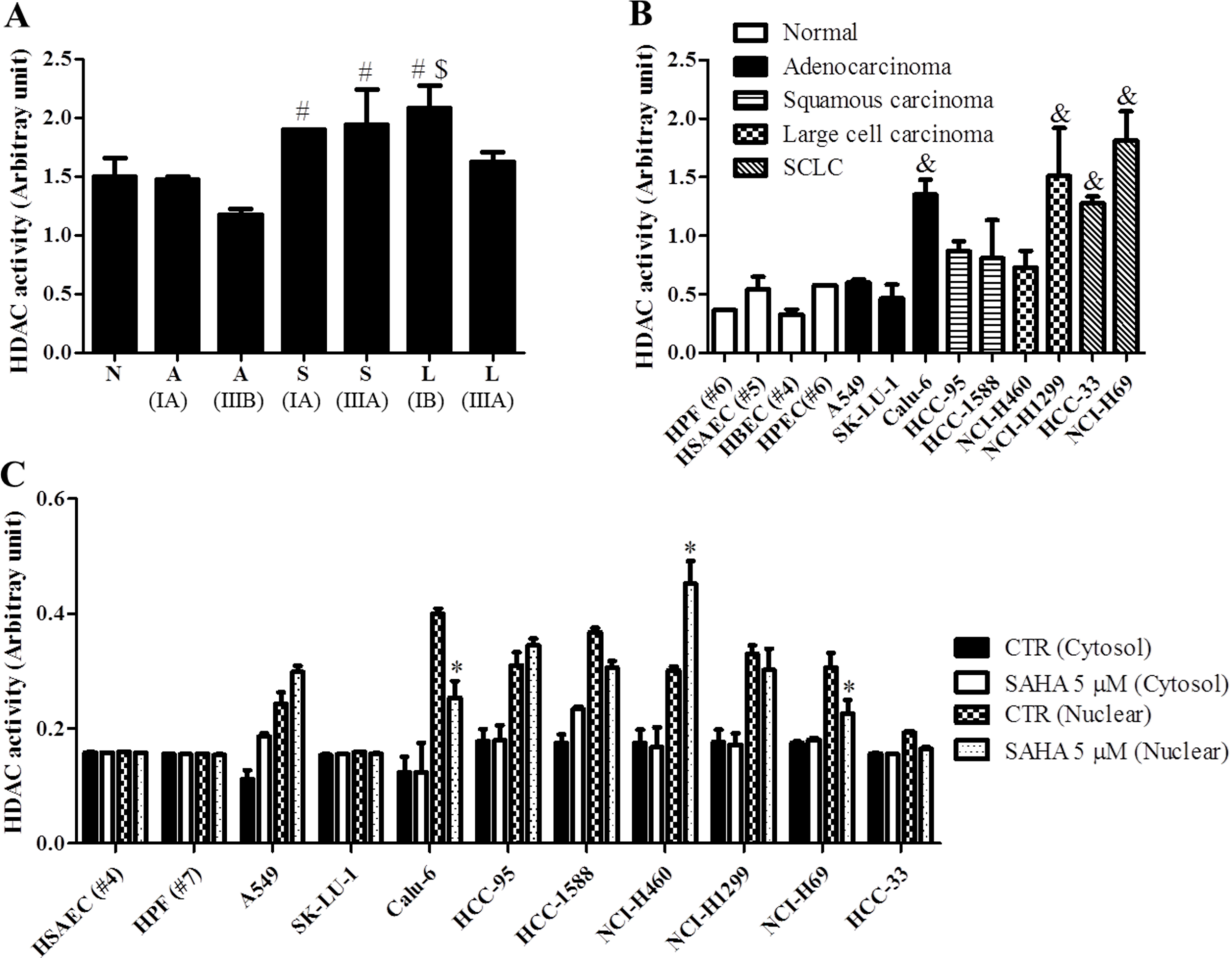
Effect of SAHA on HDAC activities in human normal lung and cancer cells (**A**) and (**B**) Graphs show the basal HDAC activities of lung cancer patients (A), normal lung and cancer cells (B). (**C**) Graph shows the cytosol and nuclear HDAC activities in normal lung and cancer cells. ^#^*p <* 0.05 compared with A (IA). ^$^*p <* 0.05 compared with A (IIIA). ^&^*p <* 0.05 compared with HPF cells. *p < 0.05 compared with SAHA-untreated control group.

Firstly, we measured the basal HDAC activities in normal lung and cancer tissues from patients. The HDAC activities of squamous cell carcinoma and large cell carcinoma were higher than that of normal and adenocarcinoma tissues (Figure 1A). In addition, stage III tissues of adenocarcinoma and large cell carcinoma showed low HDAC activities rather than stage I tissues of those (Figure 1A). It was also observed that Calu-6, NCI-H1299 and SCLC cells showed higher HDAC activities compared with normal lung and other lung cancer cells (Figure 1B).

Next, we treated with 5 μM SAHA to normal lung and cancer cells. When we measured the HDAC activities in cytosol and nuclear fraction, SAHA significantly decreased the HDAC activities of nuclear fraction in Calu-6 and NCI-H69 cells (Figure 1C). However, this agent increased the cytosol and nuclear HDAC activities of some NSCLC cells (Figure 1C).

### Effects of SAHA on cell growth and cell death in normal lung and cancer cells

**Figure 2:**
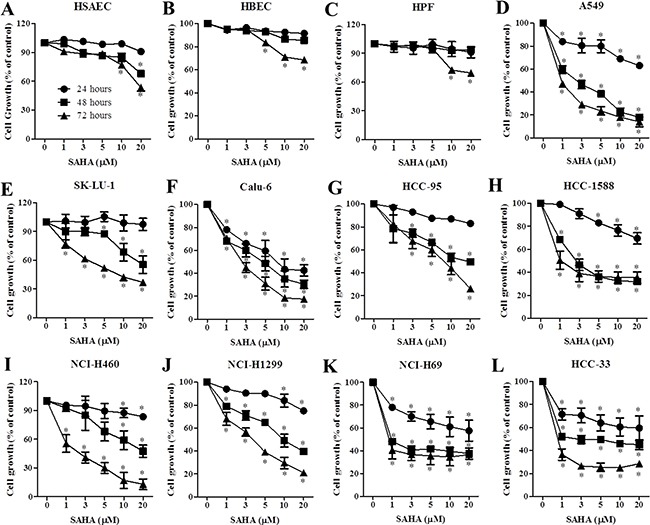
Effects of SAHA on cell growth in normal lung and cancer cells Exponentially growing cells were treated with indicated concentrations of SAHA for 24, 48 and 72 hours. Graphs show cell growth in HSAEC (**A**), HBEC (**B**), HPF (**C**), A549 (**D**), SK-LU-1 (**E**), Calu-6 (**F**), HCC-95 (**G**), HCC-1588 (**H**), NCI-H460 (**I**), NCI-H1299 (**J**), NCI-H69 (**K**) and HCC-33 (**L**). *p < 0.05 compared with SAHA-untreated control group.

SAHA did not alter the growth of normal lung, HSAEC, HBEC and HPF cells at 24 and 48 hours (Figure 2A–2C). However, SAHA inhibited the growth of lung cancer cells in dose and time-dependent manners at these times (Figure 2D–2L). Calu-6 cells were most sensitive to SAHA with an IC_50_ of 5 μM at 24 hours (Figure 2F). The IC_50_ values of SAHA in A549, HCC-1588, NCI-H69, HCC-33 cells were approximately 20 μM at 24 hours (Figure 2D, 2H, 2K, 2L). Although SK-LU-1, HCC-95, NCI-H460 and NCI-H1299 cells showed resistance to SAHA at 24 hours, SAHA dramatically decreased the growth of these cells at 48 and 72 hours (Figure 2E, 2G, 2I and 2J). This agent also inhibited normal lung cell growth at 72 hours (Figure 2A–2C). However, the susceptibility of lung cancer cells to SAHA was higher than that of normal lung cells at 72 hours.

**Figure 3:**
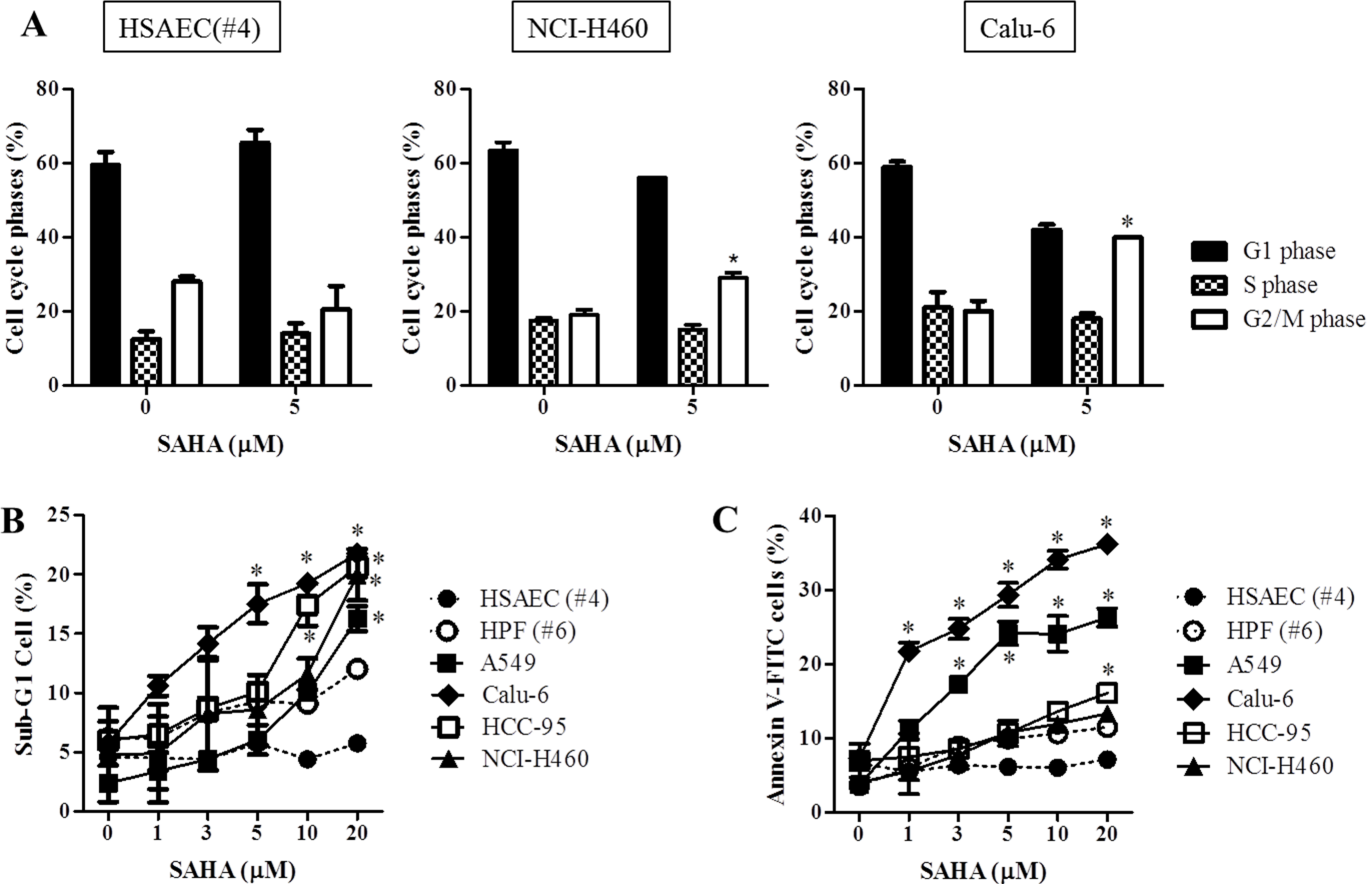
Effects of SAHA on cell cycle and cell death in normal lung and cancer cells Exponentially growing cells were treated with indicated concentrations of SAHA for 24 hours. (**A**) Graphs show the cell cycle distributions in HSAEC (#4), NCI-H460 and Calu-6 cells. (**B**) and (**C**) Graphs show the percent of sub-G1 (B) and annexin V-FITC positive cells (C). *p < 0.05 compared with SAHA-untreated control group.

When we analyzed the cell cycle phase in 5 μM SAHA-treated normal lung and cancer cells, SAHA induced a G2/M phase arrest in NCI-H460 and Calu-6 cells at 24 hours (Figure 3A). In addition, we observed that this agent led to a G2/M phase arrest in A549, SK-LU-1, HCC-95, HCC-1588 and NCI-H1299 cells (Supplementary Figure 1). However, this drug did not show any cell cycle arrest in HSAEC and HPF cells (Figure 3A and Supplementary Figure 1). Furthermore, SAHA increased sub-G1 cells and triggered apoptosis in lung cancer cells at 24 hours (Figure 3B, 3C and Supplementary Figure 2A). In HSAEC, HPF and HBEC cells, SAHA did not increase sub-G1 cells and annexin V-FITC positive cells (Figure 3B, 3C and Supplementary Figure 2A).

### Effects of SAHA on mitochondrial membrane potential, apoptosis-related protein levels and caspase activation in normal lung and cancer cells

**Figure 4:**
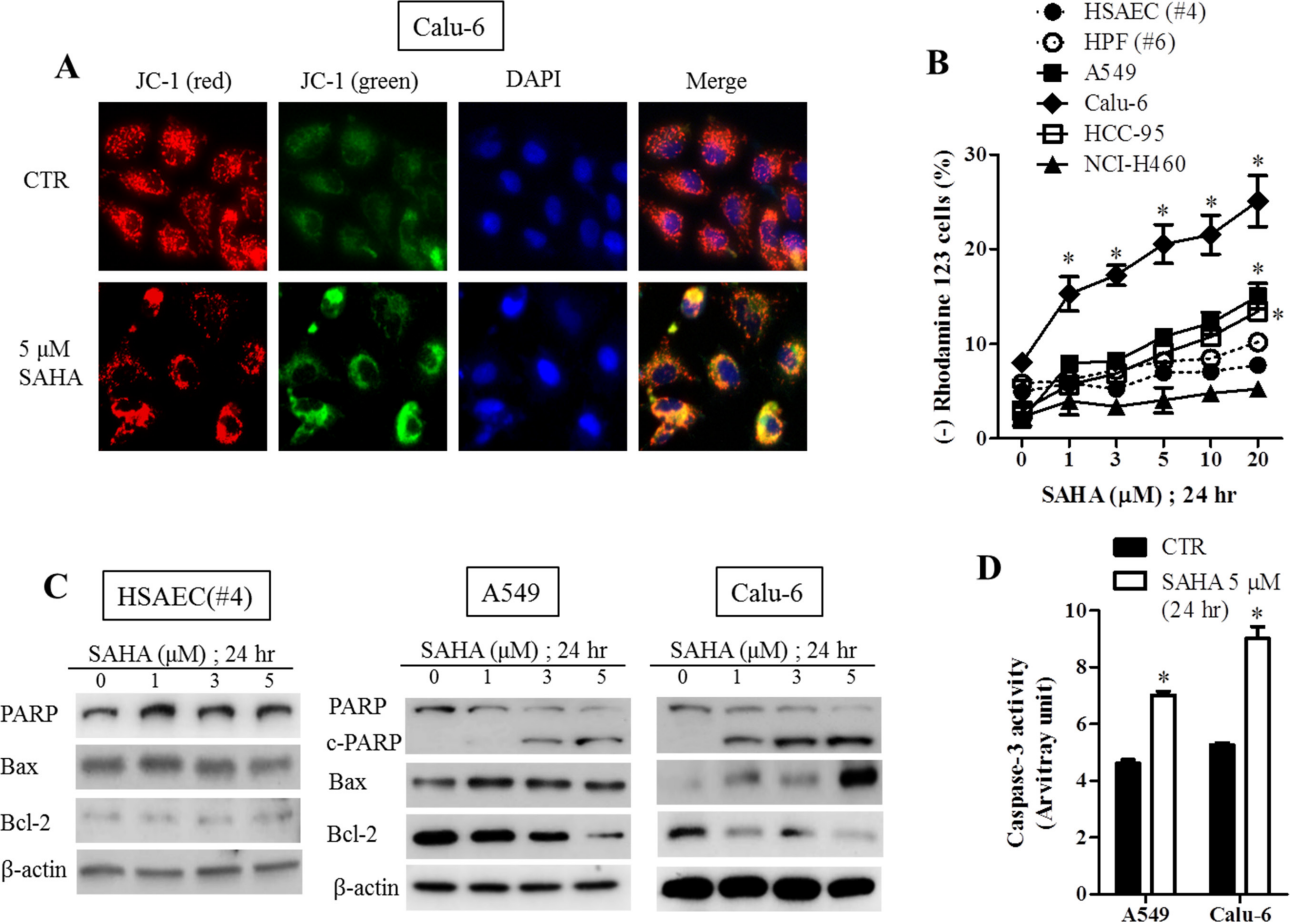
Effects of SAHA on mitochondrial membrane potential, apoptosis-related protein levels and caspase-3 activities in normal lung and cancer cells Exponentially growing cells were treated with indicated concentrations of SAHA for 24 hours. (**A**) Representative images of JC-1 red, JC-1 green, DAPI (blue) and merge in SAHA-treated Calu-6 cells. Red fluorescent images indicate high MMP (ΔΨm). Green fluorescent images shows low ΔΨm. (**B**) Graph shows the rhodamine 123 negative (ΔΨm loss) cells. (**C**) The protein levels of PARP-1, c-PARP-1, Bax, Bcl-2 and β-actin in HSAEC (#4), A549 and Calu-6 cells. (**D**) Graph shows the caspase-3 activities in A549 and Calu-6 cells. *p < 0.05 compared with SAHA-untreated control group.

SAHA increased MMP (ΔΨ_m_) loss in A549, Calu-6 (Figure 4A and 4B), HCC-33 and NCI-H69 cells (Supplementary Figure 2B). While SAHA slightly increased the loss of MMP (ΔΨ_m_) in HCC-95 and HCC-1588 cells, this agent did not affect MMP (ΔΨ_m_) in HSAEC, HPF, HBEC, SK-LU-1, NCI-H460 and NCI-H1299 cells (Figure 4B and Supplementary Figure 2B). In regard to apoptosis-related protein levels, the intact of poly (ADP-ribose) polymerase (PARP) was decreased and the cleavage for of PARP was induced by SAHA in lung cancer cells (Figure 4C and Supplementary Figure 2C). In addition, the levels of Bax were increased in SAHA-treated A549 and Calu-6 cells whereas the levels of Bcl-2 were decreased in A549, Calu-6, HCC-33 and NCI-H69 cells (Figure 4C and Supplementary Figure 2C). SAHA did not alter the levels of PARP, Bax and Bcl-2 in HSAEC (Figure 4C).

**Figure 5:**
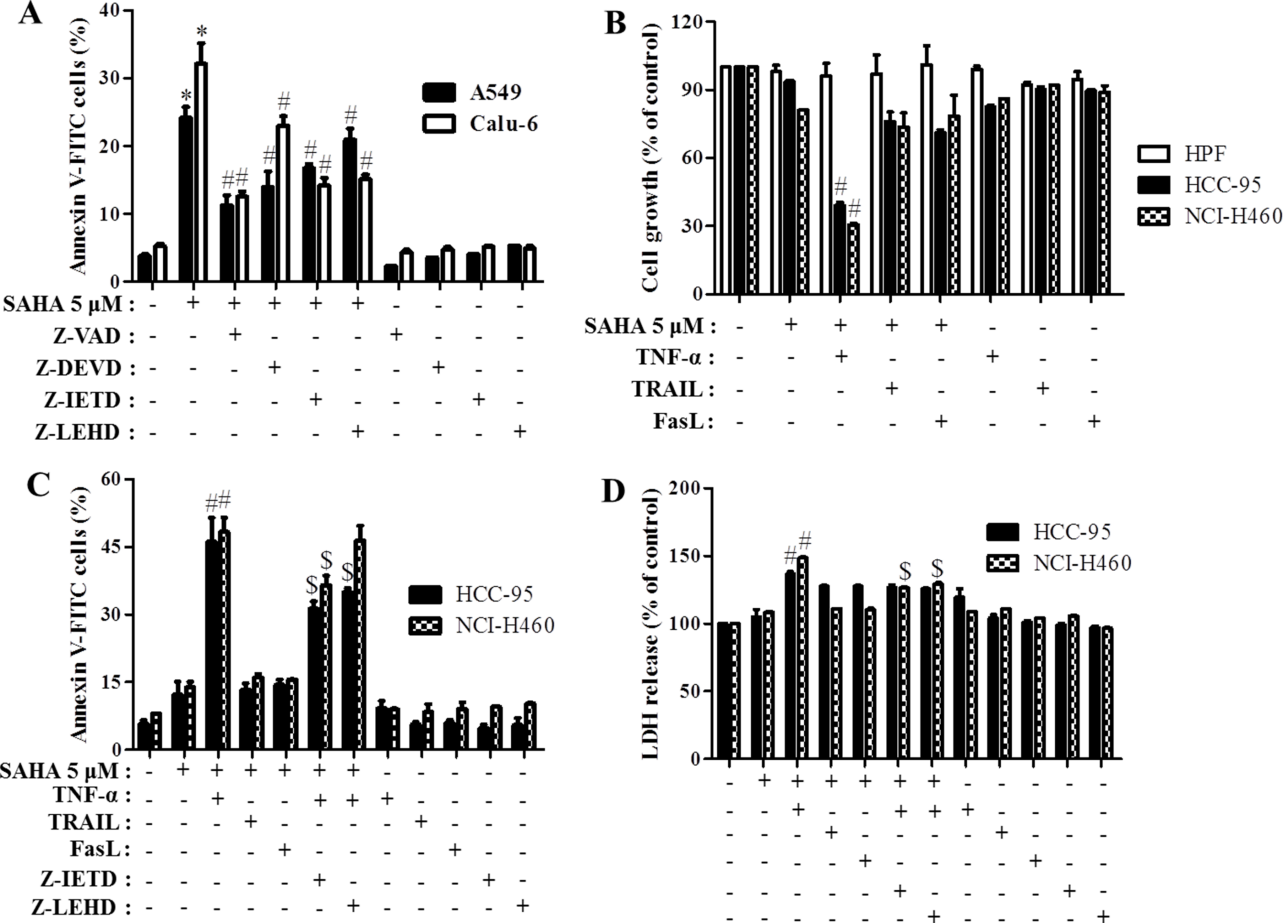
Effects of caspase inhibitors and TNF-family cytokines on cell growth and cell death in SAHA-treated normal lung and cancer cells Exponentially growing cells were treated with 5 μM SAHA and 15 μM each caspase inhibitor, 10 ng/ml TNF-α, 10 ng/ml TRAIL and 10 ng/ml FasL for 24 hours. (**A**) and (**B**) Graphs show the percent of annexin V-FITC positive cells (A) and cell growth (B). (**C**) and (**D**) Graphs show the percent of annexin V-FITC positive cells (C) and LDH release compared with that in the control cells (D). *p < 0.05 compared with SAHA-untreated control group. ^#^*p <* 0.05 compared with cells treated with SAHA only.

To determine which caspase was involved in SAHA-induced apoptosis, A549 and Calu-6 cells were pre-treated each caspase inhibitor, Z-VAD (pan-caspase inhibitor), Z-DEVD (caspase-3 inhibitor), Z-IETD (caspse-8 inhibitor) and Z-LEHD (caspase-9 inhibitor) for 1 hour prior to 5 μM SAHA treatment. We observed that SAHA increased caspase-3 activities in A549 and Calu-6 cells at 24 hours (Figure 4D). Moreover, all tested caspase inhibitors blocked apoptotic cell death caused by SAHA in A549 and Calu-6 cells (Figure 5A).

### Effects of TNF-family cytokines on apoptosis in SAHA-treated lung cancer cells

Next, to evaluate the synergistic anti-cancer effect of TNF family cytokines in lung cancer cells, we treated with 10 ng/ml TNF-α, 10 ng/ml TRAIL and 10 ng/ml FasL in HCC-95 and NCI-H460 cells. Treatment with 5 μM SAHA did not inhibit cell growth in HPF, HCC-95 and NCI-H460 cells (Figure 5B). Although all TNF-family cytokines did not alter cell growth in SAHA-treated HPF cells, only TNF-α reduced the growth of SAHA-treated HCC-95 and NCI-H460 cells (Figure 5B). In addition, TNF-α and SAHA increased apoptotic cell death and LDH release in these cells (Figure 5C and 5D). TNF-α also enhanced apoptosis in SAHA-treated A549, SK-LU-1 and Calu-6 cells (Supplementary Figure 3A). Z-IETD, a caspase-8 inhibitor, and Z-LEHD, a caspase-9 inhibitor, blocked apoptosis and necrosis caused by TNF-α in SAHA-treated HCC-95 and NCI-H460 cells (Figure 5C and 5D). However, necrosis inhibitors, NecroX-2 and Necrostatin-1 did not recover apoptosis and necrosis in TNF-α and SAHA-treated NCI-H460 cells (Supplementary Figure 3B and 3C).

### Effects of SAHA on TNFR1 expression level in lung cancer cells

**Figure 6:**
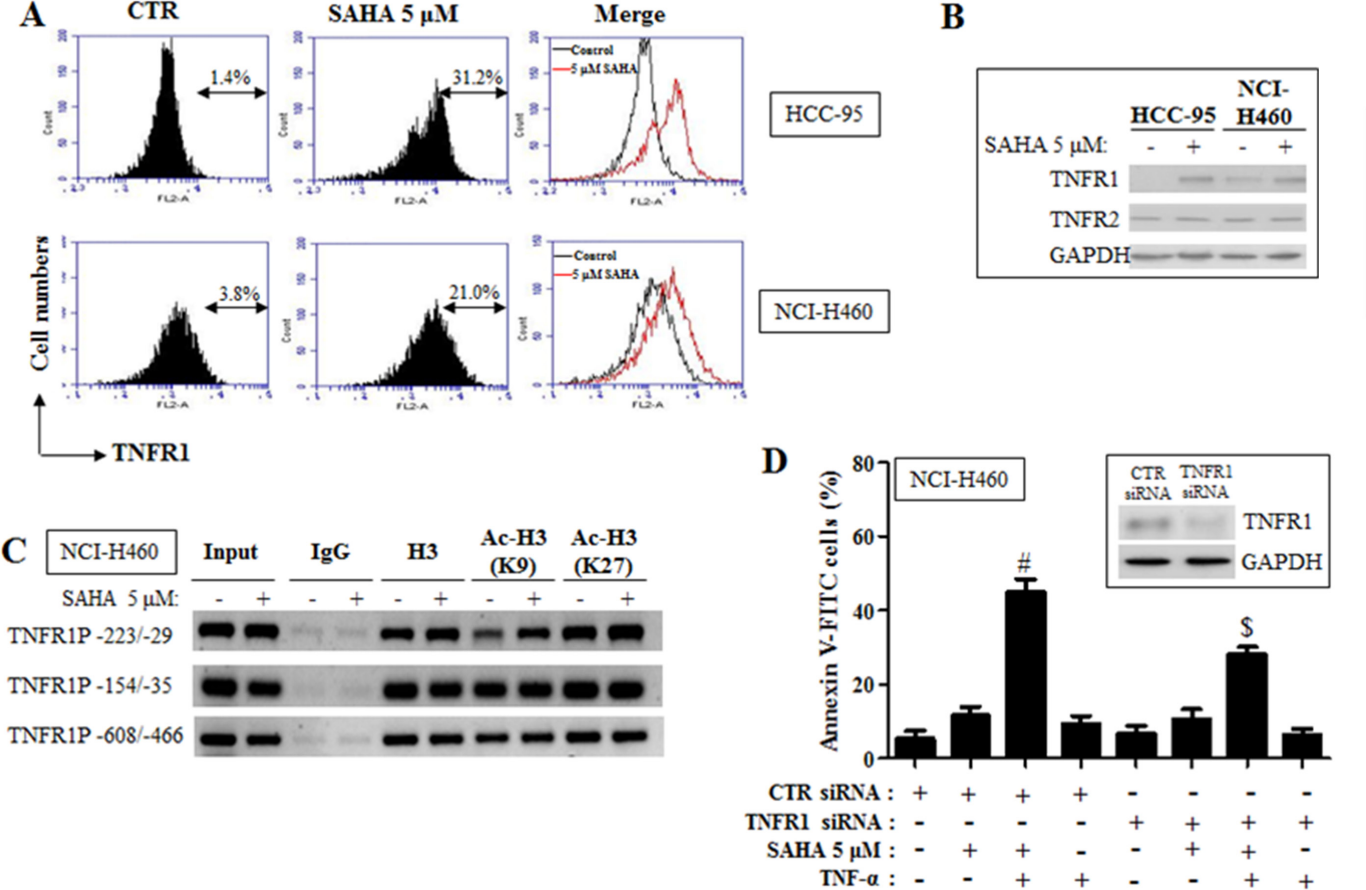
Effects of SAHA on TNFR1 expression in HCC-95 and NCI-H460 cells Exponentially growing cells were treated with 5 μM SAHA for 24 hours. (**A**) Each figure shows a representative for TNFR1 expression in HCC-95 and NCI-H460 cells. (**B**) The protein levels of TNFR1, TNFR2 and GAPDH in HCC-95 and NCI-H460 cells. (**C**) ChIP assay using H3, Ac-H3 (K9) and AC-H3 (K27) antibodies, and primers for the TNFR1 promoter regions. NCI-H460 cells were transfected with nontarget control (CTR) siRNA or TNFR1 siRNA. After one day, cells were treated with 5 μM SAHA and 10 ng/ml TNF-α for 24 hours. (**D**) Graph shows the percent of annexin V-FITC positive cells. The inside figure indicates the protein levels of TNFR1 and GAPDH in NCI-H460 cells. ^#^*p <* 0.05 compared with cells treated with SAHA only. ^$^*p <* 0.05 compared with cells treated with SAHA and TNF-α.

TNF-α regulates apoptosis, inflammation and survival through TNFR1 and TNFR2. Because TNF-α dramatically enhanced apoptotic cell death in SAHA-treated HCC-95 and NCI-H460 cells, it could be hypothesized that the level of TNFR1 or TNFR2 were increased by SAHA in these cells. As shown in Figure 6A and 6B, the expression levels of TNFR1 were increased in SAHA-treated HCC-95 and NCI-H460 cells. However, SAHA did not change the protein levels of TNFR2 in these cells (Figure 6B). SAHA also increased the TNFR1 expression level in A549 cells (Supplementary Figure 4A and 4B). Furthermore, acetylation of the region of TNFR1 promoter −223/-29 was increased in SAHA-treated NCI-H460 cells (Figure 6C). To determine whether TNFR1 level really affect apoptosis in NCI-H460 cells, these cells were transfected with TNFR1 siRNA. Administration of TNFR1 siRNA decreased apoptotic cell death induced by TNF-α and SAHA in NCI-H460 cells (Figure 6D). Similarly, TNFR1 siRNA recovered apoptosis in TNF-α and SAHA-treated A549 and SK-LU-1 cells (Supplementary Figure 4C).

## DISCUSSION

Previous studies have been demonstrated that HDAC activity is increased in many human cancers and its inhibition would be a promising therapeutic target for cancer treatment [23–25]. In fact, overexpression of HDAC2 is observed in lung cancer tissues [26]. In this study, we observed that HDAC activity of lung cancer tissues was higher than that of normal lung tissues. Likewise, lung cancer cells showed high HDAC activity rather than normal lung cells. This data implies that HDAC inhibitor can be an effective anti-cancer drug for lung cancer. In fact, there is no difference on HDAC activities between NSCLC and SCLC cells. Even though A549 and Calu-6 cells are the same adenocarcinoma cell line, the HDAC activities of these cells were different. This result indicates that HDAC activities are different depending on lung cancer cell types. SAHA as a HDAC inhibitor decreased the HDAC activity of nucleus in Calu-6 and NCI-H69 cells. However, it increased HDAC activities of nucleus and cytoplasm in some lung cancer cells. These results suggest that SAHA differently affects in normal lung and cancer cells due to different functional bioavailability in drug metabolism [27] and other HDAC might be activated as a compensatory mechanism.

As expected, SAHA inhibited the growth of lung cancer cells. We also found that SAHA decreased tumor size in lung cancer xenograft model (Supplementary Figure 5). Our results support that HDAC inhibitor show anti-cancer effect on various cancer cells including colorectal and lung [28, 29]. The susceptibility of lung cancer cells to SAHA was higher than that of normal lung cells. Some NSCLC cells showed resistance to SAHA at 24 hours. However, these cell growths were completely inhibited at 48 and 72 hours. It seems that there are no significant association between HDAC activity and SAHA resistance in lung cancer cells. These results imply that SAHA can inhibit cell growth without the inhibitory effect on HDAC. SAHA induced a G2/M phase arrest in lung cancer cells. This result supports that HDAC inhibitors triggered to a G2/M phase arrest in many cancer cells such as ovarian and pancreatic cancer [30, 31]. SAHA also increased the sub-G1 cells, annexin V-FITC positive cells and MMP (ΔΨ_m_) loss cells at 24 hours in lung cancer cells. In addition, this agent increased cleavage of PARP, Bax and cacspase-3 activity whereas it decreased Bcl-2 in lung cancer cells.

Apoptosis occurs through two pathways, mitochondria-mediated intrinsic pathway and death receptor-mediated extrinsic pathway [32]. It has been reported that HDAC inhibitors induce both intrinsic pathway and extrinsic pathway in cancer cells such as leukemia [33–35]. Similarly, SAHA led to apoptosis mediated by intrinsic pathway and extrinsic pathway because all caspase inhibitors prevented A549 and Calu-6 cell death.

TNF-family contains 19 ligands and 29 receptors in human [36]. TNF family ligands and receptors play an important role in numerous biological processes including apoptosis and inflammation [36]. There are three main cytokines, TNF-α, TRAIL and FasL. When TNF-α binds to TNFR1, it induces caspase-8 activation and then finally causes apoptosis [17]. TNF-α also activates NF-κB pathway and AP-1 signaling for cell proliferation via binding to TNFR2 [18]. TRAIL and FasL also affect apoptosis mediated by their receptors [19]. Some reports demonstrate that HDAC inhibitor and TRAIL co-treatment sensitizes cancer cells to cell death [20, 37]. In this study, only TNF-α synergistically intensified cell death in SAHA-treated lung cancer cells. Especially, TNF-α sensitized HCC-95 and NCI-H460 cells to cell death. Co-treatment with SAHA and TNF-α led to not only necrosis but also apoptosis, which was induced by activations of caspase-8 and caspase-9. Since necrosis inhibitors, NecroX-2 and Necrostatin-1 did not alter cell death, it seemed that apoptosis is major mechanism for the suppression of cell growth in TNF-α and SAHA-treated lung cancer cells.

HDAC inhibitor can affect the level of TNF-family receptors. It has been reported that HDAC inhibitor downregulates TNFR1 in leukemia and lung cancer cells [38, 39]. It was also observed that SAHA decreased the level of TNFR1 in Calu-6 cells (Supplementary Figure 4A and 4B). However, our study showed that SAHA upregulated the expression level of TNFR1 in A549, HCC-95 and NCI-H460 cells. Furthermore, acetylation of the region of TNFR1 promoter −223/-29 was increased in SAHA-treated NCI-H460 cells. This data indicates that SAHA regulates the transcription of TNFR1 in NCI-H460 cells. Administration of TNFR1 siRNA recovered apoptotic cell death in TNF-α and SAHA-treated lung cancer cells. This result suggests that up-regulation of TNFR1 by SAHA enhances the sensitivity of TNF-α to lung cancer cells. Therefore, this result demonstrates that TNF-α can be a noble combination agent for HDAC inhibitor-based cancer therapy.

**Figure 7:**
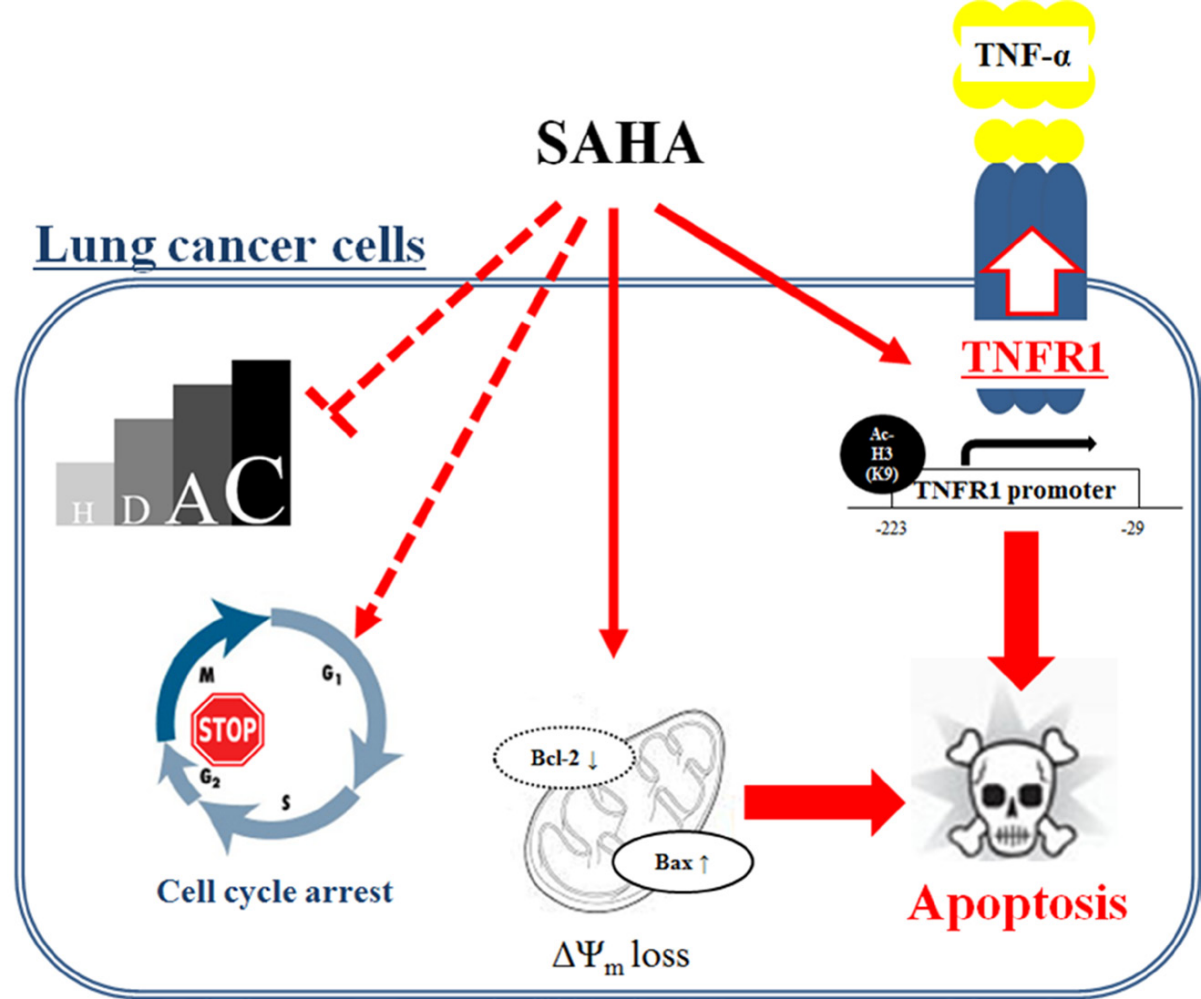
Schematic diagram of SAHA-induced cell death in lung cancer cells

In conclusion, SAHA inhibited the growth of lung cancer cells via a G2/M phase arrest and caspase-dependent apoptosis regardless of the inhibitory effect on HDAC. SAHA also enhanced apoptotic effect of TNF-α in human lung cancer cells through up-regulation of TNFR1 (Figure 7). These results suggest that TNF-α may be a key to improve the anti-cancer effect of HDAC inhibitors in lung cancer.

## MATERIALS AND METHODS

### Cell culture

The human lung adenocarcinoma A549, SK-LU-1, Calu-6, squamous cell carcinoma HCC-95, HCC-1588, large cell carcinoma NCI-H460, NCI-H1299, SCLC HCC-33 and NCI-H69 cells were obtained from the American Type Culture Collection (ATCC, Manassas, VA). Human small airway epithelial cells (HSAEC), human bronchial epithelial cells (HBEC), human pulmonary artery endothelial cells (HPEC) and human pulmonary fibroblast (HPF) cells were obtained from PromoCell GmbH (Heidelberg, Germany). These cells were maintained in incubator containing 5% CO_2_ at 37°C. Normal lung and cancer cells were cultured RPMI-1640 containing 10% fetal bovine serum (FBS, Sigma-Aldrich Co., St. Louis, MO) and 1% penicillin-streptomycin (Gibco BRL, Grand Island, NY). Cells were grown in 100 mm plastic cell culture dishes (BD Falcon. Franklin Lakes, NJ) and harvested with a trypsin-EDTA (Gibco BRL). HSAEC, HBEC, HPEC and HPF cells were used between passages four to seven.

### Reagents

Protein lysates of normal lung tissue, adenocarcinoma tissues, squamous cell carcinoma tissues and large cell carcinoma tissues were purchased from OriGene Technology (Rockville, MD) (Supplementary Table 1). SAHA from Cayman Chemical Company (Ann Arbor, MI) was dissolved in dimethyl sulfoxide (DMSO; Sigma-Aldrich Co.) at 10 mM as a stock solution. The pan-caspase inhibitor (Z-VAD-FMK; benzyloxycarbonyl-Val-Ala-Asp-fluoromethylketone), caspase-3 inhibitor (Z-DEVD-FMK; benzyloxycarbonyl-Asp-Glu-Val-Asp-fluoromethylketone), caspase-8 inhibitor (Z-IETD-FMK; benzyloxycarbonyl-Ile-Glu-Thr-Asp-fluoromethylketone) and caspase-9 inhibitor (Z-LEHD-FMK; benzyloxycarbonyl-Leu-Glu-His-Asp-fluoromethylketone) were obtained from R&D Systems, Inc. (Minneapolis, MN) and were dissolved in DMSO at 10 mM to serve as stock solutions. TNF-α, TRAIL and FasL were also obtained from R&D systems, Inc. and were dissolved in water at 10 mg/ml as a stock solution. NecroX-2 and Necrostatin-1 from Enzo Life Sciences (Plymouth Meeting, PA) were dissolved in DMSO at 1 mM and 50 mM as a stock solution, respectively. Cells were pretreated with each caspase inhibitor for 1 hour prior to SAHA treatment. DMSO (0.01%) was used as a control-vehicle and it did not affect cell growth and death.

### Measurement of HDAC activity

The HDAC activity was measured by using the HDAC assay kit, according to manufacturer's instructions (Millipore, Billerica, MA). Briefly, 1 × 10^6^ cells in 60 mm culture dish (BD Falcon) were incubated with or without 5 μM SAHA for 24 hours. Then cells were washed with PBS and added in 4 volumes of lysis buffer (R&D systems, Inc.). Thirty μg of total protein were used to measure the HDAC activity. These were added to each well in 96-well microtiter plates (SPL Life Sciences, Pocheon, Gyeonggi-do, Korea) with HDAC substrate provided from the kit at 37°C for 1 hour. The optical density of each well was measured at 405 nm by using a microplate reader (Synergy™ 2, BioTekR Instruments Inc., Winooski, VT).

### Cell growth inhibition assay

The effect of SAHA on growth inhibition in normal lung and cancer cells was determined by the 3-(4,5-dimethylthiazol-2-yl)-2,5-diphenyltetrazolium bromide (MTT, Sigma-Aldrich Co.) assay. Briefly, 5 × 10^3^ cells in 96-well microtiter plate (SPL Life Sciences) were incubated with the indicated concentrations of SAHA with or without TNF-α, TRAIL or FasL for indicated times. Then twenty microliter of MTT solution [2 mg/mL in phosphate-buffered saline (PBS)] was added to each well in 96-well plates. The plates were incubated for 4 hours at 37^°^C. Medium in plates was removed by pipetting, and 200 μl DMSO was added to each well to solubilize the formazan crystals. The optical density was measured at 570 nm using a microplate reader (Synergy™ 2, BioTekR Instruments Inc.).

### Cell cycle and sub-G1 analysis

Cell cycle and sub-G1 analysis were determined by propidium iodide (PI, Sigma-Aldrich Co.; Ex/Em = 488 nm/617 nm) staining. Briefly, 1 × 10^6^ cells in 60 mm culture dish (BD Falcon) were incubated with the indicated concentrations of SAHA for 24 hours. Cells were washed with PBS and then incubated with 10 μg/ml PI with RNase at 37^°^C for 30 min. Cell cycle distribution and sub-G1 DNA content cells were measured and analyzed with an Accuri C6 flow cytometer (BD Sciences, Franklin Lakes, NJ).

### Detection of apoptosis

Apoptosis was detected by staining cells with annexin V-fluorescein isothiocyanate (FITC, Life Technologies, Carlsbad, CA ; Ex/Em = 488 nm/519 nm). Briefly, 1 × 10^6^ cells in 60 mm culture dish (BD Falcon) were incubated with the indicated concentrations of SAHA with or without each caspase inhibitor, TNF-α, TRAIL or FasL for 24 hours. Then cells were washed twice with cold PBS and added 500 μl of binding buffer (10 mM HEPES/NaOH pH 7.4, 140 mM NaCl, 2.5 mM CaCl_2_) at a concentration of 1 × 10^6^ cells/ml. Five microliters of annexin V-FITC and PI were added to these cells, which were analyzed with an Accuri C6 flow cytometer (BD Sciences).

### Measurement of MMP (ΔΨm)

MMP (ΔΨm) levels were measured using rhodamine 123 (Sigma-Aldrich Co.; Ex/Em = 485 nm/535 nm) and JC-1 dyes (Enzo Life Sciences ; Ex/Em = 515 nm/529 nm). Briefly, 5 × 10^4^ cells in 12 well culture plate (BD Falcon) were incubated with the indicated concentration of SAHA for 24 hours. Cells were washed twice with PBS and incubated with 0.1 μg/ml rhodamine 123 or 10 μg/ml JC-1 at 37^°^C for 30 min. For staining nucleus, cells were incubated with 500 nM 4′, 6′-diamidino-2-phenylindole (DAPI, Life Technologies, Ex/Em = 358 nm/461 nm) at 37^°^C for 30 min. The intensity of rhodamine 123 staining was determined by Accuri C6 flow cytometry (BD Sciences). The negative staining of rhodamine 123 from cells indicated the loss of MMP (ΔΨm) in cells. After incubation with JC-1 and DAPI, cells were washed three times with PBS and images were collected by using a fluorescence microscope (FLoid^®^ Cell Imaging Station, Life Technologies) in × 400 magnification. Green fluorescence indicates a monomer at low ΔΨm and red fluorescence presents high ΔΨm.

### Western blotting

The protein expression levels were evaluated by Western blotting. Briefly, 1 × 10^6^ cells in 60 mm culture dish (BD Falcon) were incubated with the indicated concentrations of SAHA for 24 hours. Then cells were washed with PBS and added in 4 volumes of lysis buffer (Intron Biotechnology, Seongnam, Gyeonggi-do, Korea). Thirty μg of total protein were resolved by 4–20% SDS-PAGE gels, and then transferred to Immobilon-P PVDF membranes (Millipore) by electroblotting. Then membranes were probed with anti-PARP, anti-c-PARP, anti-Bax, anti-Bcl-2, anti-TNFR2 (Cell signaling Technology, Danvers, MA), anti-TNFR1, anti-β-actin and anti-GAPDH (Santa Cruz Biotechnology, Santa Cruz, CA). Membranes were incubated with horseradish peroxidase-conjugated secondary antibodies. Blots were developed using an EZ-Western Lumi Pico ECL solution kit (DoGen, Seoul, Korea).

### Measurement of caspase-3 activity

The caspase-3 activity was assessed by using the caspase-3 colorimetric assay kit (R&D systems, Inc.) Briefly, 1 × 10^6^ cells in 60 mm culture dish (BD Falcon) were incubated with 5 μM SAHA for 24 hours. Then cells were washed with PBS and added in 4 volumes of lysis buffer (R&D systems, Inc.). Fifty μg of total protein were used to measure the caspase-3 activity. These were added to each well in 96-well microtiter plates (SPL Life Sciences) with DEVD-pNA as a caspase-3 substrate. The plates were incubated for 1 hour at 37^°^C. The optical density of each well was measured at 405 nm by using a microplate reader (Synergy™ 2).

### Lactate dehydrogenase (LDH) release assay

Necrosis in cells treated with SAHA and/or TNF family cytokines was evaluated by LDH kit (Sigma-Aldrich Co.) Briefly, 1 × 10^6^ cells in 60 mm culture dish (BD Falcon) were incubated with the indicated concentrations of SAHA with or without TNF family cytokines for 24 hours. After treatment, the cell culture media were collected and centrifuged for 5 min at 1500 rpm. Fifty μl of the media supernatant was added to a 96 well plate along with LDH assay reagent and then incubated at room temperature for 30 min. The absorbance values were measured at 490 nm using a microplate reader (Synergy™ 2). LDH release was expressed as the percentage of extracellular LDH activity compared with the control cells.

### Measurement of TNFR1 expression by using flow cytometer

Surface expression of TNFR1 was detected by PE conjugated TNFR1 antibody (Santa Cruz Biotechnology). PE conjugated Armenian hamster IgG (Santa Cruz Biotechnology) was used as an isotype control. Briefly, 5 × 10^4^ cells in 12 well culture plate (BD Falcon) were incubated with the indicated 5 μM SAHA for 24 hours. Then cells were washed twice with cold PBS and added 500 μl of PBS at a concentration of 1 × 10^6^ cells/ml. Ten microliters of PE conjugated TNFR1 antibodies or five microliters of PE conjugated Armentian hamster IgG were added to these cells, which were analyzed with an Accuri C6 flow cytometer (BD Sciences).

### Chromatin immunoprecipitation (ChIP) assay

ChIP assay was performed using SimpleChIP Enzymatic Chromatin IP Kit (#9003 Cell signaling Technology) according to manufacturer's instructions. Briefly, 1 × 10^6^ cells in 60 mm culture dish (BD Falcon) were incubated with 5 μM SAHA for 24 hours. Cells are fixed with 1% formaldehyde to cross-link and then DNA is digested with Micrococcal Nuclease for 20 min at 37^°^C. The digested DNA incubated overnight at 4^°^C with 1 μg of following antibody: rabbit IgG, anti-H3 (1b2b2), anti-AcH3(K9) and anti-AcH3 (K27) (Cell signaling Technology). Immunoprecipitated samples were eluted using protein G magnetic beads, crosslinks reversed and then DNA purified according to manufacturer's instruction. PCR was performed using specific primers for the regions of the TNFR1 promoter, TNFR1-223 to −29 (forward 5′- GAT TGGTGGGTTGGGGGCACA and reverse 5′- ATT AAAGCAGAGAGGAGGGGAGAGA), TNFR1-154 to −35 (forward 5′- AGTTAAAGAACGTTGGGCCTCCT and reverse 5′- GCAGAGAGGAGGGGAGAGAAGG) and TNFR1-608 to −466 (forward 5′- TTCCCAAGAAA GAGGGAGACTAGGA and reverse 5′- CTGGGGTTC CTGTAAGGATTTGTTC). PCR products were run on 2% agarose gel.

### Transfection of cells with TNFR1 siRNA

Gene silencing of TNFR1 was performed by using siRNA. A nonspecific control (CTR) siRNA duplex [5′-CCUACGCCACCAAUUUCGU(dTdT)-3′] and TNFR1 siRNA duplex [5′-CCUGGACAAGCACAUAGCA(dTdT)-3′] were purchased from the Bioneer Corp. (Daejeon, South Korea). In brief, 2.5 × 10^5^ cells in six-well plates (Nunc) were incubated in RPMI-1640 supplemented with 10% FBS. The next day, cells (approximately 30–40% confluence) in each well were transfected with the CTR or TNFR1 siRNA duplex [80 pmol in Opti-MEM (GIBCO BRL)] using LipofectAMINE 2000 (Invitrogen, Brandford, CT). One day later, cells were treated with or without 5 μM SAHA and 10 ng/ml TNF-α for additional 24 hours. The transfected cells were used for Western blot analysis and annexin V-FITC staining measurements.

### Statistical analysis

The results represent the mean of at least three independent experiments (mean ± SD). The data were analyzed using Instat software (GraphPad Prism5, San Diego, CA). The Student's *t-test* or one-way analysis of variance (ANOVA) with post hoc analysis using Tukey's multiple comparison test was used for parametric data. Statistical significance was defined as *p <* 0.05.

## SUPPLEMENTARY MATERIALS FIGURES AND TABLE



## Abbreviations

SAHA: suberoylanilide hydroxamic acid
HDAC: histone deacetylase
TNF: tumor necrosis factor
TRAIL: TNF-related apoptosis-inducing ligand
FasL: Fas ligand
TNFR: TNF receptor
NSCLC: non-small cell lung cancer
SCLC: small cell lung cancer
HSAEC: human small airway epithelial cells
HBEC: human bronchial epithelial cells
HPEC: human pulmonary artery endothelial cells
HPF: human pulmonary fibroblast
FBS: fetal bovine serum
MTT: 3-(4,5-dimethylthiazol-2-yl) −2,5-diphenyltetrazolium bromide
PI: propidium iodide
FITC: fluorescein isothiocyanate
Z-VAD-FMK: benzyloxycarbonyl-Val-Ala-Asp-fluoromethylketone
Z- DEVD-FMK: benzyloxycarbonyl-Asp-Glu-Val-Asp-fluoromethylketone
Z-IETD-FMK: benzyloxycarbonyl-Ile-Glu-Thr-Asp-fluoromethylketone
Z-LEHD-FMK: benzyloxycarbonyl-Leu-Glu-His-Asp-fluoromethylketone
MMP (ΔΨm): mitochondrial membrane potential
DAPI: 4’, 6’-diamidino-2-phenylindole
LDH: lactate dehydrogenase
ChIP: chromatin immunoprecipitation
