# Supplementary material for: Suberoylanilide hydroxamic acid increases anti-cancer effect of tumor necrosis factor-α through up-regulation of TNF receptor 1 in lung cancer cells
Source: Oncotarget. 2017 Jan 13;8(11):17726–37. doi: 10.18632/oncotarget.14628 (PMC5392281; doi:10.18632/oncotarget.14628)
Supplement: Supplementary file 1 [file oncotarget-08-17726-s001.pdf]

Suberoylanilide hydroxamic acid increases anti-cancer effect of tumor necrosis factor-α through up-regulation of TNF receptor 1 in lung cancer cells

Supplementary Materials

Supplementary Table 1: Information about normal lung and cancer tissues from patients

Type	Stage	Gender	Age	OriGene ID
Normal	–	Caucasian female	55	CP565391
Adecarcinoma	I A	Female	61	CP565398
Adecarcinoma	III B	Caucasian female	69	CP565411
Squamous carcinoma	I A	Female	72	CP565792
Squamous carcinoma	III A	Female	76	CP565564
Large cell carcinoma	I B	Caucasian female	79	CP565586
Large cell carcinoma	III A	Female	56	CP565534

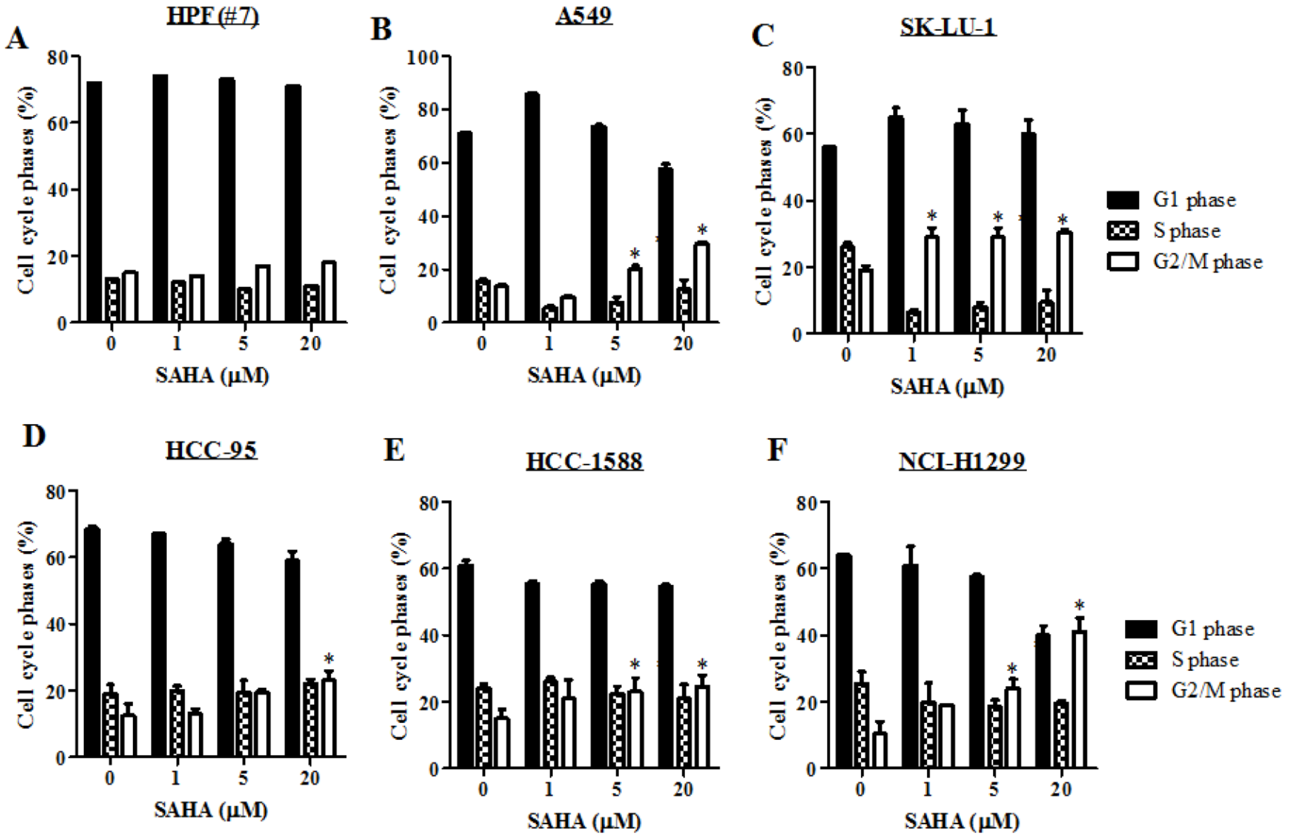

Supplementary Figure 1: Effects of SAHA on cell cycle in normal and lung cancer cells. Exponentially growing cells were treated with indicated concentrations of SAHA for 24 hours. (**A**–**F**) Graphs show the cell cycle distributions in HPF (#7) (A), A549 (B), SK-LU-1 (C), HCC-95 (D), HCC-1588 (E) and NCI-H1299 cells (F). *$p < 0.05$ compared with SAHA-untreated control group.

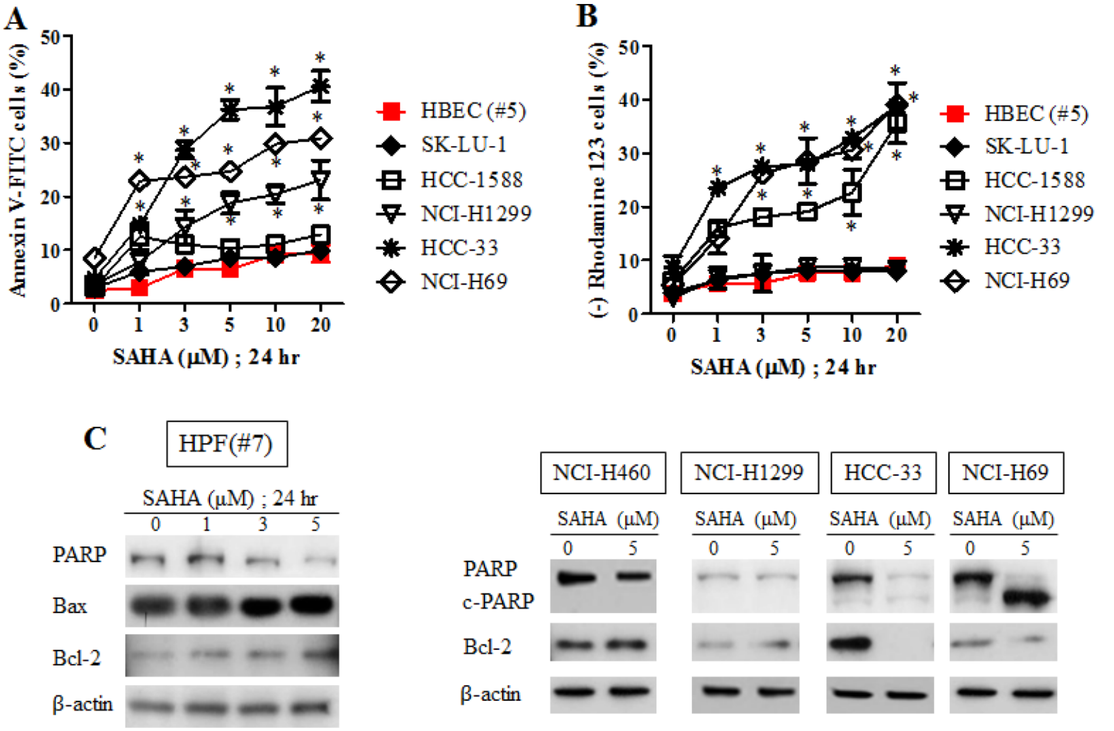

Supplementary Figure 2: Effects of SAHA on cell death, mitochondrial membrane potential and apoptosis-related protein levels in normal lung and cancer cells. Exponentially growing cells were treated with indicated concentrations of SAHA for 24 hours. (**A** and **B**) Graphs show the annexin V positive cells (A) and the rhodamine 123 negative (ΔΨm loss) cells (B). (**C**) The protein levels of PARP-1, c-PARP-1, Bax, Bcl-2 and β-actin in HPF (#7), NCI-H460, NCI-H1299, HCC-33 and NCI-H69 cells. *$p < 0.05$ compared with SAHA-untreated control group.

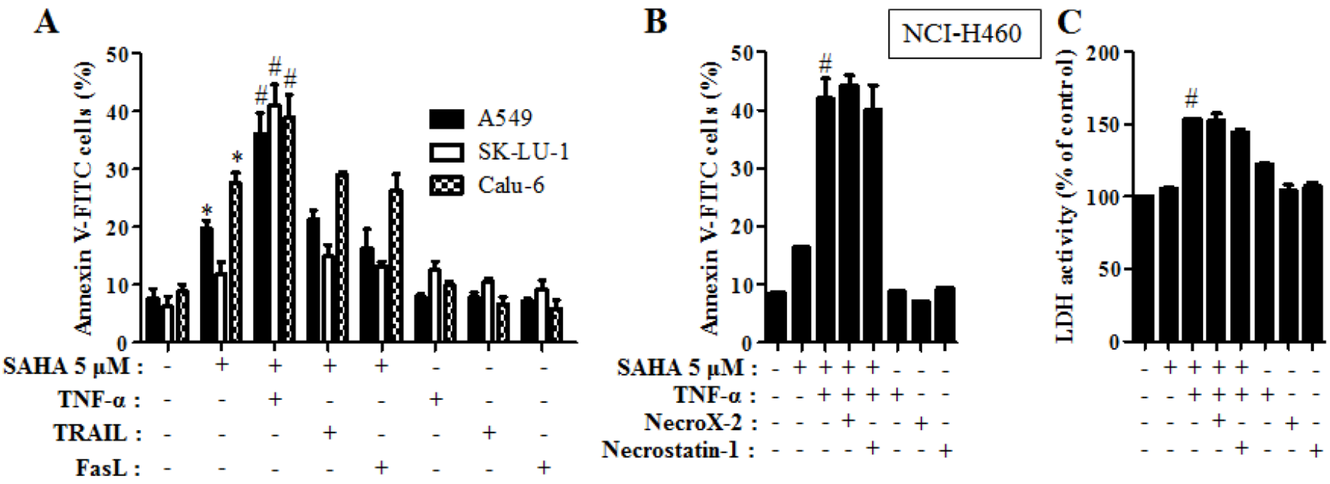

Supplementary Figure 3: Effects of TNF family cytokines on apoptosis and necrosis in SAHA-treated lung cancer cells. Exponentially growing cells were treated with 5 μM SAHA, 10 ng/ml TNF-α, 10 ng/ml TRAIL, 10 ng/ml FasL, 1 μM NecroX-2 and 50 μM Necrostatin-1 for 24 hours. (**A** and **B**) Graphs show the percent of annexin V-FITC positive cells. C: Graph shows the LDH release compared with that in the control cells. *$p < 0.05$ compared with SAHA-untreated control group. $^{\#}p < 0.05$ compared with cells treated with SAHA only.

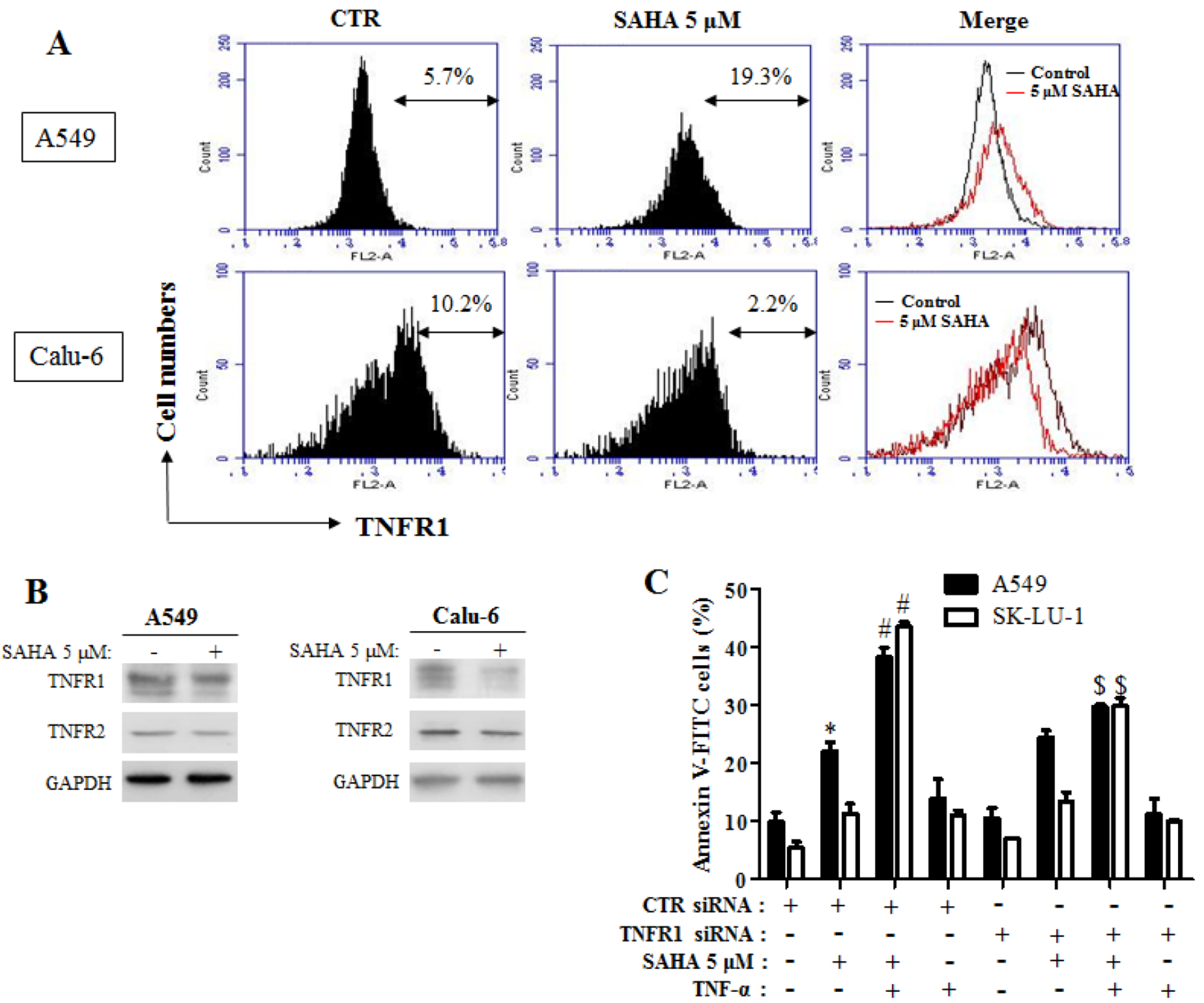

Supplementary Figure 4: Effects of SAHA on TNFR1 expression in lung cancer cells. Exponentially growing cells were treated with 5 μM SAHA for 24 hours. A549 and SK-LU-1 cells were transfected with nontarget control (CTR) siRNA or TNFR1 siRNA. After one day, cells were treated with 5 μM SAHA and 10 ng/ml TNF-α for 24 hours. (**A**) Each figure shows a representative for TNFR1 expression in A549 and Calu-6 cells. (**B**) The protein levels of TNFR1, TNFR2 and GAPDH in A549 and Calu-6 cells. (**C**) Graph shows the percent of annexin V-FITC positive cells. *$p < 0.05$ compared with SAHA-untreated control group. $^{\#}p < 0.05$ compared with cells treated with SAHA only. $^{\$}p < 0.05$ compared with cells treated with SAHA and TNF-α.

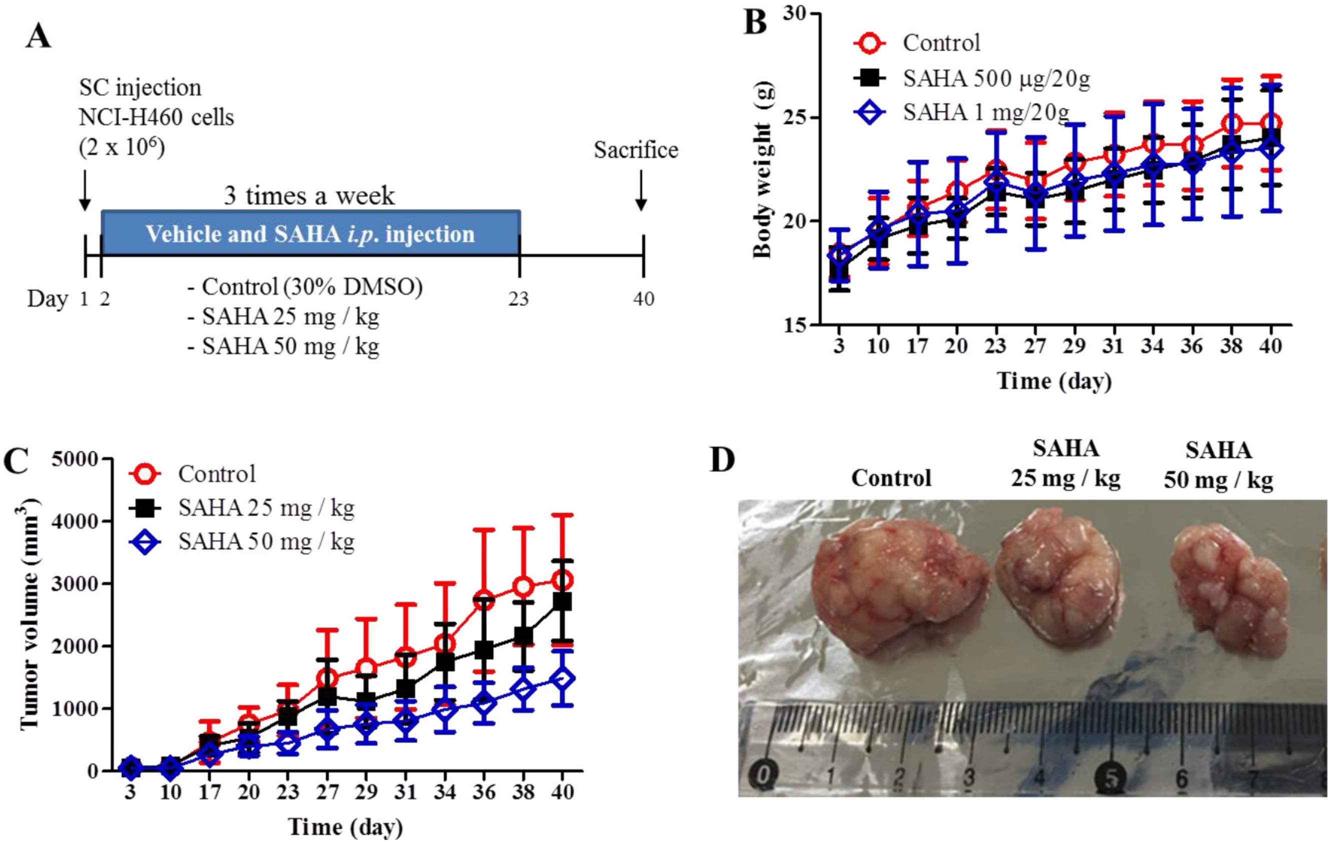

Supplementary Figure 5: Effect of SAHA on lung cancer xenograft model. Four weeks old female BALB/c-nude mice were purchased from Nara-Biotec (Seoul, South Korea). The current study protocol was approved by the Institutional Animal Care and Use Committee of Chonbuk National University. (**A**) NCI-H460 cells (2×10^6) were subcutaneously injected into the flank of 4 weeks old female nude mice to establish a tumor xenograft model. 30% DMSO (control), 25 mg/kg and 50 mg/kg SAHA were injected intraperitoneally (i.p.) three times for 23 days. On 17 days after last i.p. injection, mice were sacrificed and tumors were extracted. Each group included 4 mice. (**B** and **C**) Graphs show body weight (B) and tumor volume (C). (**D**) Image of the representative tumors of control, 25 mg/kg and 50 mg/kg SAHA injected group.
